# Isolation, Chemical Structure, and Antagonistic Activity Against Galectin‐1 of Water‐Soluble Polysaccharides From *Plantago asiatica*

**DOI:** 10.1002/fsn3.70562

**Published:** 2025-07-01

**Authors:** Yiqing Li, Yonghong Ma, Guanyu Li, Yanqing Jia, Chengxin Sun, Liushaoqiu Zhou, Wenqin Lei, Tao Zhang

**Affiliations:** ^1^ Department of Laboratory Medicine Affiliated Hospital of Zunyi Medical University Zunyi China; ^2^ School of Laboratory Medicine Zunyi Medical University Zunyi China; ^3^ School of Pharmacy Zunyi Medical University Zunyi Guizhou China

**Keywords:** cervical cancer, chemical structure, Galectin‐1, *Plantago asiatica*, polysaccharide, purification

## Abstract

*Plantago asiatica* has been utilized as a dietary supplement in health foods and for medicinal purposes, enjoying a long‐standing acceptance throughout history. A neutral polysaccharide fraction (WPA‐N) and three acidic polysaccharide fractions (WPA‐1, WPA‐2, and WPA‐3) prepared from *Plantago asiatica* by water extraction and DEAE‐cellulose were tested for their chemical structures and inhibitory effects on galectin‐1‐mediated bioactivity. The results showed that WPA‐N was composed of Glc and Gal residues, while WPA‐1, WPA‐2, and WPA‐3 were mainly constituted by GalA, Gal, Ara, Rha, as well as some other monosaccharide residues, with the molecular weights ranging from 68.7 kDa to 168.2 kDa. Galectin‐1 has been identified as a mediator in the multi‐step process of tumor cell aggregation, migration, and invasion, as well as in tumor‐induced angiogenesis, including in cervical cancer. Except for WPA‐N, three acidic fractions could inhibit galectin‐1‐mediated hemagglutination and also could inhibit the fluorescence intensity of galectin‐1 protein, and WPA‐3 showed the strongest activity. Besides, anti‐cancer experiments showed three acidic fractions could inhibit the growth and migration of cervical cancer ME180 cells, and WPA‐3 inhibited the proliferation and migration activity of ME180 better than others. Biolayer interferometry analysis found that WPA‐3 had a very strong binding ability to galectin‐1 protein, with a *KD* value of 26.8 nM. WPA‐3 contained rhamnogalacturonan‐I mainchain with branched galactan, arabinan, and arabinogalactans‐II side chains and some homogalacturonan domain. These findings indicated the potential applications in functional food fields of *Plantago asiatica* polysaccharides.

## Graphical abstract

*Plantago asiatica* has been utilized as a dietary supplement in health foods and for medicinal purposes, enjoying a long‐standing acceptance throughout history. This study will provide new insights into the relationship between the antagonistic activity against galectin‐1 of *P.asiatica* polysaccharides and their applications in the fields of food and medicine.

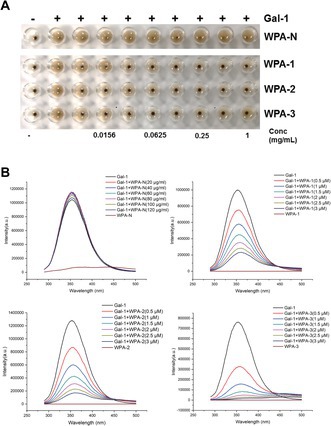

## 1 Introduction

*Plantago asiatica* L. ( *P. asiatica* ), widely planted in China and some Asian countries, has been used as dietary supplement products and functional foods owing to the good ingredients (Ji, Hou, and Guo 2019; Li, Wang, et al. 2020). In China, *P. asiatica* can cure nephropathy, cough, pain, etc. In other Asian countries, *P. asiatica* can be used to treat inflammatory bowel disease and skin trauma owing to its anti‐inflammatory, antiseptic, antibacterial effects, etc. (Akbar 2020; Belorio and Gómez 2021). In the functional food industry fields, some studies reported that *P. asiatica* products displayed significant biological activity with increasing satiety, regulating blood glucose and lipid concentration, and promoting urination and defecation (Brum et al. 2016; Lertpipopmetha et al. 2019; Zhang, Hu, et al. 2021, Zhang, Qiao, et al. 2021).

Polysaccharides are one of the main components of *P. asiatica* , besides other active compounds including flavonoids, phenolic acids, iridoid glycosides, and alkaloids (Ji, Hou, and Guo 2019; Ji, Zhang, et al. 2019; Samuelsen 2000; Zhang, Hu, et al. 2021, Zhang, Qiao, et al. 2021). Polysaccharides, complex biomacromolecular polymers composed of several types of monosaccharide residues, are widely produced by plants, microorganisms, and animals, and have been applied in the food fields for a long time (Azeredo and Waldron 2016; Cazón et al. 2017; Ji et al. 2023). A wide variety of biological activities of polysaccharides have been reported, including antitumor, anti‐inflammatory, antioxidant, immunomodulatory, and antidiabetic activities (Tian et al. 2024; Tzianabos 2000; Yu et al. 2018). Some studies have found that the *P. asiatica* polysaccharide and its purified fractions exhibit wide biological activities, such as immunomodulatory, anti‐inflammatory, and hepatoprotective activities (Hu et al. 2018; Ji, Hou, and Guo 2019; Ji, Zhang, et al. 2019; Li, Du, et al. 2020; Li, Huang, et al. 2020; Li, Wang, et al. 2020; Niu et al. 2017). However, until now, the antagonistic activity against galectin‐1 (Gal‐1) of *P. asiatica* polysaccharide is little studied. Our preliminary experiment results found that *P. asiatica* polysaccharide had the ability to inhibit the activity of Gal‐1. Gal‐1 is a homodimeric protein with 14 kDa subunits and is the prototype of the Galectin superfamily, known for its strong binding to β‐galactosides via a conserved carbohydrate‐recognition domain. In cancers like cervical, lung, melanoma, prostate, ovarian, thyroid, pancreatic, head–neck, bladder, uterine, and colorectal cancers, Gal‐1 is frequently overexpressed and plays a role in cancer progression by interacting with glycoconjugates within the tumor microenvironment. By targeting Gal‐1 and its role in normalizing blood vessels and enhancing the immune response against tumors, multiple pathways may be addressed, potentially increasing the tumor's sensitivity to medication. Therefore, creating effective Gal‐1 inhibitors represents a promising strategy for cancer treatment (Jonathan and Mary 2016; Astorgues‐Xerri et al. 2014). In this study, four polysaccharide fractions were purified from *P. asiatica*, and the primary structures and inhibitory effects on Gal‐1‐mediated bioactivity were studied. These findings will provide new insights into the relationships between the antagonistic activity against Gal‐1 of *P. asiatica* polysaccharides and their applications in the field of functional foods.

## 2 Material and Methods

### 2.1 Reagents and Consumables

The aerial parts of *P. asiatica* were purchased from Qingdao City, Shandong Province, China. 1‐phenyl‐3‐methyl‐5‐pyrazolone (PMP), standard dextrans (10–2000 kDa), and galactose (Gal), arabinose (Ara), fucose (Fuc), glucuronic acid (GlcA), xylose (Xyl), galacturonic acid (GalA), rhamnose (Rha), mannose (Man), and glucose (Glc) were obtained from Sigma (Darmstadt, Germany). DEAE‐cellulose material in the chromatographic column was obtained from Shanghai Chemical Reagent (Shanghai, China). PrimeSTAR HS DNA Polymerase, Taq DNA Polymerase, *Nde I*, and *Xho I* were purchased from TaKaRa Company (Dalian, China). GoldView nucleic acid dye and isopropyl‐β‐**D**‐thiogalactoside (IPTG) were purchased from Solarbio Life Sciences Company (Beijing, China). Human Gal‐1 cDNA ORF Clone in Cloning Vector was purchased from Sino Biological Company (Beijing, China). The other analytical‐grade reagents were all obtained from China.

### 2.2 Preparation of Recombinant Human Gal‐1

Plasmid pCMV3‐SP‐N‐His Vector (Sino Biological, China) containing the full sequence of human Gal‐1 was used as a PCR template. The forward primer was 5′‐CGCCATATGATGGCTTGTGGTCTGGTCGC‐3′, and the reverse primer was 5′‐CCGCTCGAGTCAGTCAAAGGCCACACATT‐3′. The expression plasmids for Gal‐1 were constructed by inserting its cDNA into the vector pET‐15b between the *Nde I* and *Xho I* cut sites and confirmed by DNA sequencing and cleavage of restriction endonuclease. An overnight incubation with 0.25 mM IPTG at 25°C transformed and induced *E. coli BL21 (DE3)* cells to express Gal‐1. Gal‐1 protein was purified with lactosyl‐Sepharose CL‐6B according to the published method (Zhou et al. 2020), and SDS‐PAGE was used to analyze the purity of Gal‐1.

### 2.3 Preparation and Purification of *P. asiatica* Polysaccharide

In the same conditions, 14 L of distilled water was used twice to extract 500 g of the dry *P. asiatica* . Filtrates were concentrated to 700 mL, and up to 75% ethanol was added to aqueous filtrates to precipitate polysaccharides from *P. asiatica* (WPA), which were then lyophilized by vacuum freeze dryers. A DEAE‐cellulose column (40 cm × 4.5 cm) was loaded with the supernatant of the WPA (distilled in dH_2_O). The column was eluted with dH_2_O followed by 0.1 M, 0.2 M, 0.3 M, 0.5 M NaCl solution stepwise to give four polysaccharide fractions (WPA‐N, WPA‐1, WPA‐2, WPA‐3) monitored by the phenol–sulfuric acid method (Dubois et al. 1956).

### 2.4 Monosaccharide Composition Determined of WPA Fractions

Methanol containing 2 M HCl was applied to each of the WPA fractions (2 mg) for 16 h at 80°C, and then hydrolyzed with distilled water containing 2 M TFA at 120°C for 1 h. Hydrolyzates were treated with PMP, and the products were detected by the Waters e2695 system coupled with a Waters 2489 UV detector and a Dikma Platisil ODS column (250 mm × 4.6 mm). An elution rate of 1.0 mL/min was used with 82% PBS (pH 7.0, 0.1 M) and 18% acetonitrile (v/v), monitored at 245 nm during the elution (Zhang, Zu, et al. 2020; Zhang, Liu, et al. 2020; Zhang, Shuai, et al. 2020).

### 2.5 Molecular Weight Determined of WPA Fractions

The average molecular weight of WPA fractions was analyzed by the published method. In brief, WPA fractions (5 mg/mL for each) were analyzed by a Shimadzu HPLC coupled with a Schambeck RI2000A refractive index detector (RID) and a TSKgel G4000PWxl column (7.8 mm × 300 mm, TOSOH, Japan) precalibrated with standard dextrans. At a flow rate of 0.5 mL/min, the column was eluted with 0.2 M NaCl and monitored by RID. The linear regression equation was used to calculate the molecular weights of the WPA fractions (Zhang, Zu, et al. 2020; Zhang, Liu, et al. 2020; Zhang, Shuai, et al. 2020).

### 2.6 FT‐IR Spectra Determined of WPA Fractions

For FT‐IR (Bruker Tensor 27) analysis, each fraction of WPA was mixed with KBr powder and pressed into a 1 mm pellet. The spectra were recorded at the frequency range of 4000–400 cm^−1^.

### 2.7 NMR Spectra Determined of WPA‐3

WPA‐3 (25 mg) was dissolved in 0.5 mL D_2_O (99.8%), and its one‐dimensional NMR spectra (^1^H, ^13^C) and two‐dimensional NMR spectra (HSQC) were recorded by a Bruker AV600 spectrometer (Karlsruhe, Germany).

### 2.8 Gal‐1‐Mediated Hemagglutination Assay

According to the published protocol, a Gal‐1‐mediated hemagglutination experiment was performed to analyze the inhibitory activity of WPA fractions on Gal‐1 (Shuai et al. 2024; Zhang, Zu, et al. 2020; Zhang, Liu, et al. 2020; Zhang, Shuai, et al. 2020). A transparent microplate (V type) was used in this experiment, and in each well, 25 μL of 0.15 M NaCl (control) or 25 μL of WPA fraction solution was added, along with 25 μL of 1% bovine serum albumin (PBS, pH 7.4), 25 μL of 2 μg/mL Gal‐1 solution (PBS, pH 7.4), and 25 μL of 4% (V/V) chicken erythrocyte suspension (PBS, pH 7.4). The concentration of WPA fraction that completely inhibited the aggregation of chicken erythrocytes was recorded after incubating for 0.5 h at room temperature, and a minimum inhibitory concentration (MIC) was shown to describe its inhibitory activity on Gal‐1.

### 2.9 Fluorescence Spectra Determined of WPA Fractions

When the excitation wavelength is set at 280 nm, the emission spectrum can be used for the detection of Gal‐1 (Trp residues) to analyze whether it is bound and inhibited or not (Cui et al. 2004; Zhang et al. 2008). Gal‐1 was diluted with PBS and prepared into a solution of 0.55 μM, and each of the WPA fractions was diluted from 0.5 μM to 3 μM (WPA‐1, WPA‐2, WPA‐3) or from 20 μg/mL to 120 μg/mL (WPA‐N). WPA fractions with different concentrations and Gal‐1 solution are mixed and reacted at 4°C for 3 h. These mixture solutions were detected and analyzed using a Max‐4 fluorescence spectrometer (Horiba, Japan), with the excitation wavelength set at 280 nm and emission wavelengths ranging from 290 nm to 500 nm.

### 2.10 Biolayer Interferometry (BLI) Analysis Interaction Between WPA‐3 and Gal‐1

The interaction between WPA‐3 and Gal‐1 was analyzed by a ForteBio Octet RED 96 instrument. After hydration with PBS for 10 min, the Ni‐NTA biosensors (Fortebio) were used. His‐tagged Gal‐1 was added at a concentration of 10 μg/mL, with monitoring performed as follows: loading for 180 s, the association for 120 s, and the dissociation for 180 s. To determine binding kinetics, five concentrations (40, 20, 10, 5, 2.5 μg/mL) of WPA‐3 were dissolved in PBS. Data were analyzed using ForteBio Data Analysis software for the determination of binding parameters.

### 2.11 Antitumor Activity of WPA Fractions in Vitro

#### 2.11.1 Antiproliferation Activity Assay

A humidified atmosphere containing 5% CO_2_ was used to culture the human cervical cancer cell line ME180 cells in RPMI 1640 medium (Gibco, USA) supplemented with 10% fetal bovine serum (Gibco, USA). Cell proliferation inhibitory activity was measured by the CCK‐8 assay. ME180 cells were seeded in a sterile 96‐well plate at a density of 1 × 10^4^ cells/mL (100 μL/well) and cultured in 5% CO_2_ at 37°C for 24 h. ME180 cells were then treated with or without (control group) different concentrations (0 mg/mL, 0.25 mg/mL, 0.5 mg/mL, 1 mg/mL) of WPA fractions (dissolved in culture medium) for 48 h. 110 μL CCK‐8 solution (mixed with culture medium at 1:10) was added to each well and incubated with the cells for another 2 h. The optical density was detected by a microplate reader (Thermo, USA) at 450 nm. In order to calculate the antiproliferative activity of WPA fractions, the following formula was used:

Cell viabilities (% control) = (*A*_2_–*A*_0_)/(*A*_1_–*A*_0_) × 100, where *A*_0_ is the absorbance of the well with medium but without cells; *A*_1_ is the absorbance of the well with medium and cells; *A*_2_ is the absorbance of the well with WPA fractions, medium, and cells.

#### 2.11.2 Cell Migration by Scratching Wound Assays

In 6‐well cell culture plates, ME180 cells were plated at a density of 5 × 10^5^ cells/well in RPMI 1640 growth medium containing 10% fetal bovine serum and incubated until 80% density was achieved. The cell monolayer was scratched with a vertical line with a 20 μL pipette tip. The cells were washed thrice with PBS, and WPA fractions (0.5 mg/mL, dissolved in culture medium) were added and incubated for 48 h. An inverted microscope was used to observe and photograph the migration of ME180 cells into wound areas. The initial width of the scratch was recorded (*W*_1_), and the width of the scratch after being treated with WPA fractions was recorded (*W*_2_). A control group of cells was treated in the same way as the experimental group, but only treated with medium after pretreatment and scratching. The wound closure was calculated as: wound closure (%) = (*W*_1_–*W*_2_)/*W*_1_ × 100.

#### 2.11.3 Cell Migration by Transwell Assays

Using a 24‐well Transwell plate with polycarbonate inserts of 8 μm pore size (Corning, USA), transwell assays were performed. Resuspend ME180 cells at a density of 5 × 10^5^ in 100 μL of serum‐free pretreatment medium and seed them into the upper chamber of the Transwell unit. In the lower chamber, a complete medium with or without WPA fractions of 0.5 mg/mL was used. ME180 cells were allowed to migrate at 37°C in a CO_2_ incubator for 3 days. The cells that migrated to the underside were fixed in 4.0% paraformaldehyde for 25 min at room temperature and stained in 0.1% crystal violet for 3 min. The filter was washed three times with dH_2_O for removal of excess dye. The wells were photographed by an inverted microscope, and the migrated cell counts were analyzed by Image J.

### 2.12 Statistical Analysis

The data of this study were shown as the means ± SD, and one‐way analysis of variance (ANOVA) and Duncan's multiple range test were applied to check the significance of difference at the significance level of 0.05 by using SPSS 29.0 software.

## 3 Results and Discussion

### 3.1 Preparation and Purification of WPA and Gal‐1

**FIGURE 1:**
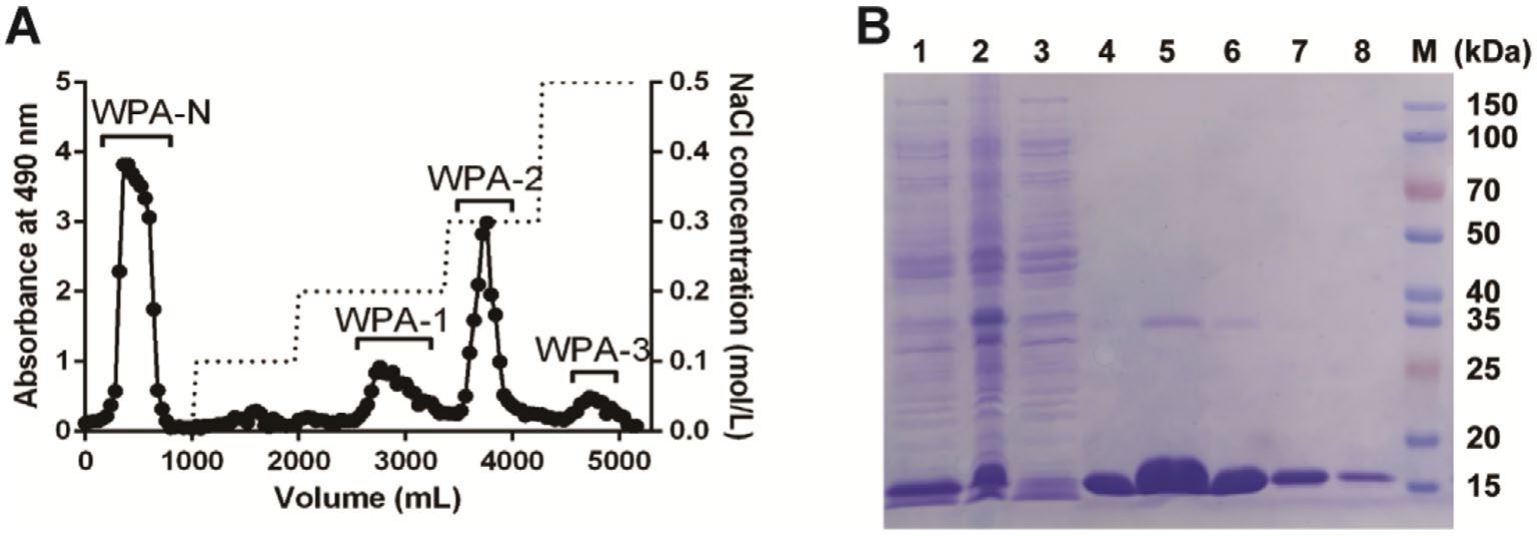
Preparation and purification of WPA and Gal‐1 protein. (A) Elution profiles of WPA on DEAE‐cellulose chromatography. (B) Purity of Gal‐1 analyzed by 12% SDS‐PAGE. Lane 1: Supernatant after bacterial lysis; Lane 2: Precipitate after bacterial lysis; Lane 3: Uninduced bacteria; Lane 4–8: Supernatant from lactosyl‐Sepharose CL‐6B column eluted by 200 mM lactose; Lane M: Maker.

The total polysaccharide of *P. asiatica* (WPA, yield 6.5% of dried material, w/w) was collected by boiling water extraction and ethanol precipitation. As shown in Figure 1A, after isolation by a DEAE‐cellulose chromatography eluted stepwise with dH_2_O, 0.1 M, 0.2 M, 0.3 M, and 0.5 M NaCl solution, four major polysaccharide fractions WPA‐N (yield 61.4% to WPA, w/w), WPA‐1 (yield 4.1% to WPA, w/w), WPA‐2 (yield 7.7% to WPA, w/w), and WPA‐3 (yield 2.4% to WPA, w/w) were collected, dialyzed, and vacuum freeze‐dried.

The human full‐length Gal‐1 plasmid was transferred into the expression strain *Escherichia coli* BL21 (DE3), and Gal‐1 protein was induced by IPTG and purified by a lactosyl‐Sepharose CL‐6B affinity chromatography which was performed based on the published method (Zhou et al. 2020). Gal‐1 protein was solubilized and expressed in the supernatant, and the purity of Gal‐1 was analyzed by 12% SDS‐PAGE, as shown in Figure 1B.

### 3.2 Monosaccharide Composition and Molecular Weight of WPA Fractions

The monosaccharide composition of WPA fractions was detected using HPLC by pre‐column derivatization method. As shown in Table 1, the results showed that the monosaccharide composition of WPA‐N was simpler than that of three acidic fractions, which were mainly composed of Glc (74.2%) and Gal (25.8%). The monosaccharide composition of WPA‐1, WPA‐2, and WPA‐3 was complex, composed of more than seven types of saccharide residues (Rha, GalA, Gal, Ara, Glc, Xyl, Man). Three acidic fractions might be pectic polysaccharides due to their high GalA content. The ratio of Rha/GalA in WPA‐1, WPA‐2, and WPA‐3 was 0.11, 0.13, and 0.56, respectively, so Rha/GalA of these three fractions was all falling within the RG‐I domain ranged from 1.0 to 0.05 reported by Schols and Voragen (1996). Additionally, Rha/GalA of these three fractions was all less than 1.0, suggesting WPA‐1, WPA‐2, and WPA‐3 probably contained some HG domains, especially for WPA‐1 and WPA‐2. WPA‐1, WPA‐2, and WPA‐3 also all contained numbers of neutral sugar residues, such as Gal, Ara, and Glc, suggesting that three acidic fractions might contain some side chains including galactan, arabinogalactan, and/or glucan attached to polysaccharide backbones.

**TABLE 1:** Yield, monosaccharide composition, molecular weight of WPA fractions.

Fraction	Yield (%W)^a^	Monosaccharide composition (mol%)							Mw (kD)
		Rha	GalA	Gal	Ara	Glc	Xyl	Man	
WPA‐N	61.4	—	—	25.8	—	74.2	—	—	21.6
WPA‐1	4.1	8.7	72.9	7.4	8.7	1.6	—	0.7	68.7
WPA‐2	7.7	8.5	65.4	6.2	6.9	4.3	7.4	1.3	168.2
WPA‐3	2.4	17.5	31.1	18.6	13.7	12.9	—	6.2	78.9

^a^ Yields in relation to WPA.

**FIGURE 2:**
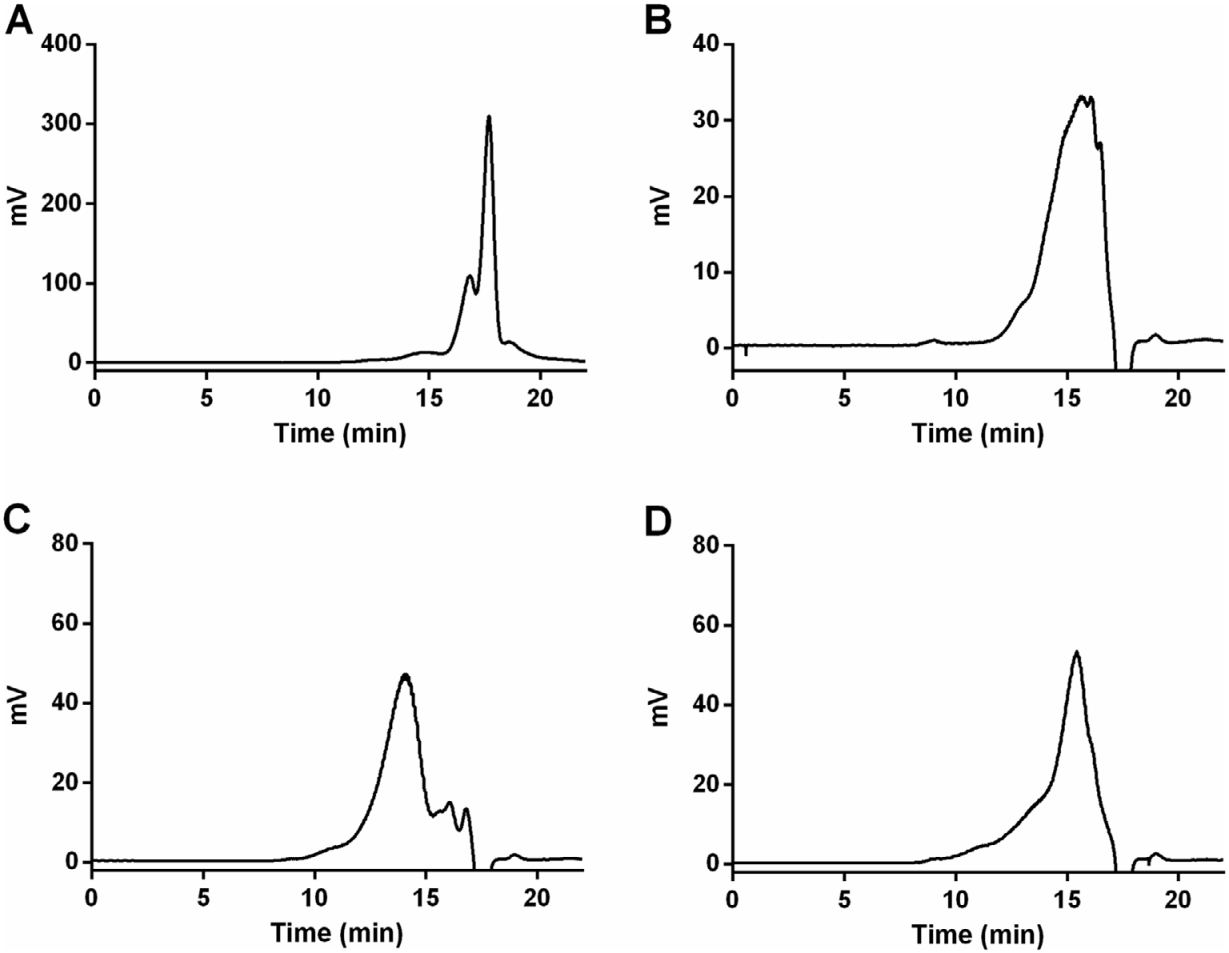
The average molecular weights of WPA‐N (A), WPA‐1 (B), WPA‐2 (C), and WPA‐3 (D). Sub‐fractions were applied to a TSKgel G4000PWxl column, which was eluted with 0.2 M NaCl at a flow rate of 0.5 mL/min.

The molecular weight of WPA fractions was analyzed by HPGPC with a TSKgel G4000PW_xl_ column. As shown in Figure 2, the major average molecular weights of WPA‐N, WPA‐1, WPA‐2, and WPA‐3 were approximately estimated to be 21.6 kDa, 68.7 kDa, 168.2 kDa, and 78.9 kDa, respectively.

### 3.3 FT‐IR Spectra of WPA Fractions

**FIGURE 3:**
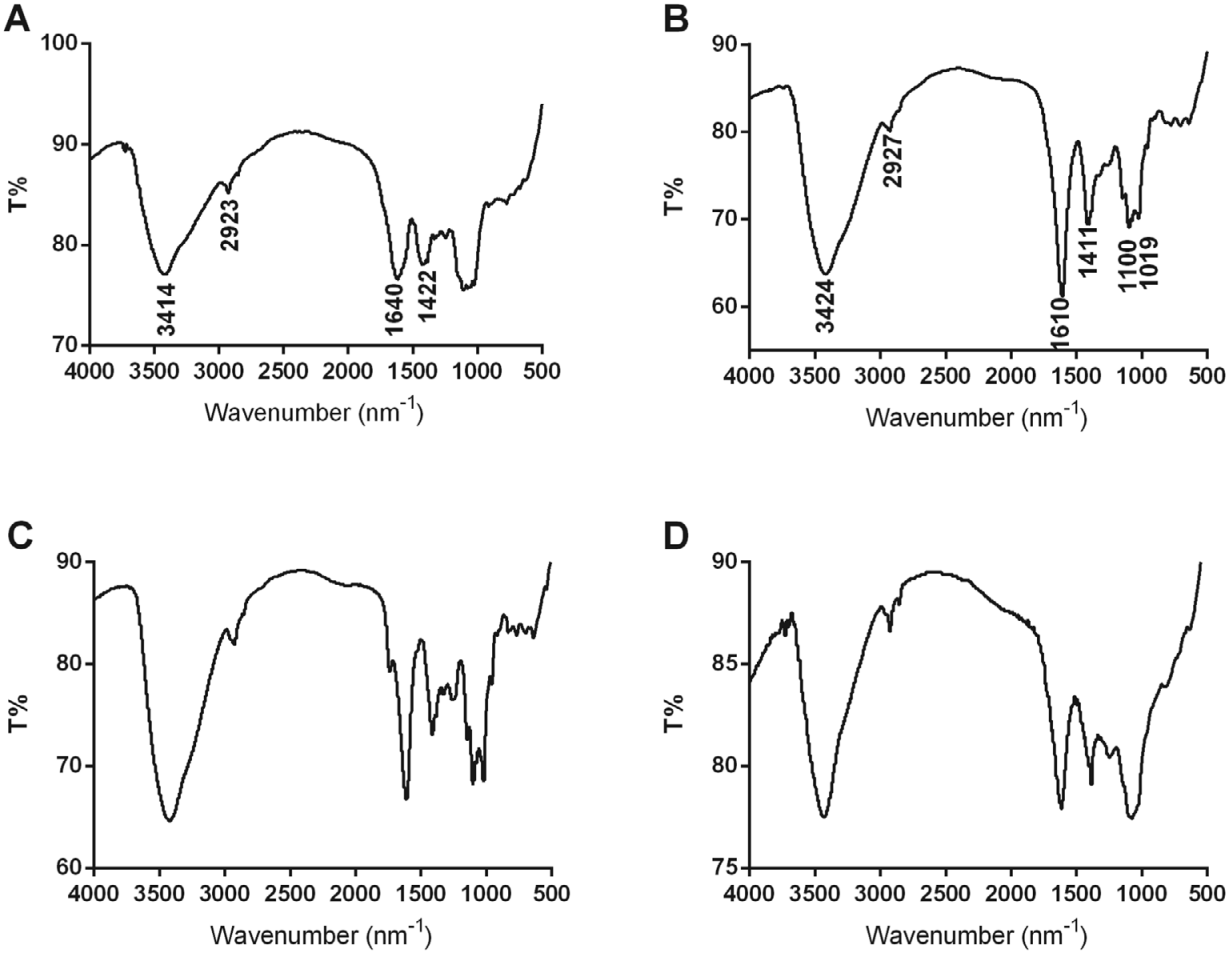
FT‐IR spectra of WPA‐N (A), WPA‐1 (B), WPA‐2 (C), and WPA‐3 (D).

FT‐IR spectrum is an important technique for primary structural property analysis of polysaccharides, which could provide a more accurate description of purified WPA fractions. As shown in Figure 3, the peaks at 3414 cm^−1^ or 3424 cm^−1^ and 2923 cm^−1^ or 2927 cm^−1^ were owing to O–H and C–H chemical bonds, respectively; the peak at 1610 cm^−1^ was owing to the C=O chemical bonds in GalA residues, and the peak at 1640 cm^−1^ was owing to the C–O chemical bonds in WPA‐N (Ji, Hou, and Guo 2019; Ji, Zhang, et al. 2019; Liang et al. 2019; Zhang, Zu, et al. 2020; Zhang, Liu, et al. 2020; Zhang, Shuai, et al. 2020). The peaks at 1019 cm^−1^ and 1100 cm^−1^ suggested that WPA‐1, WPA‐2, and WPA‐3 were all rich in uronic acid (Capek et al. 2003; Coimbra et al. 1998). The FT‐IR spectra of WPA‐1 and WPA‐3 were similar, but differed slightly in peak intensity.

### 3.4 Inhibitory Activities on Gal‐1‐Mediated Bioactivity by WPA Fractions

**FIGURE 4:**
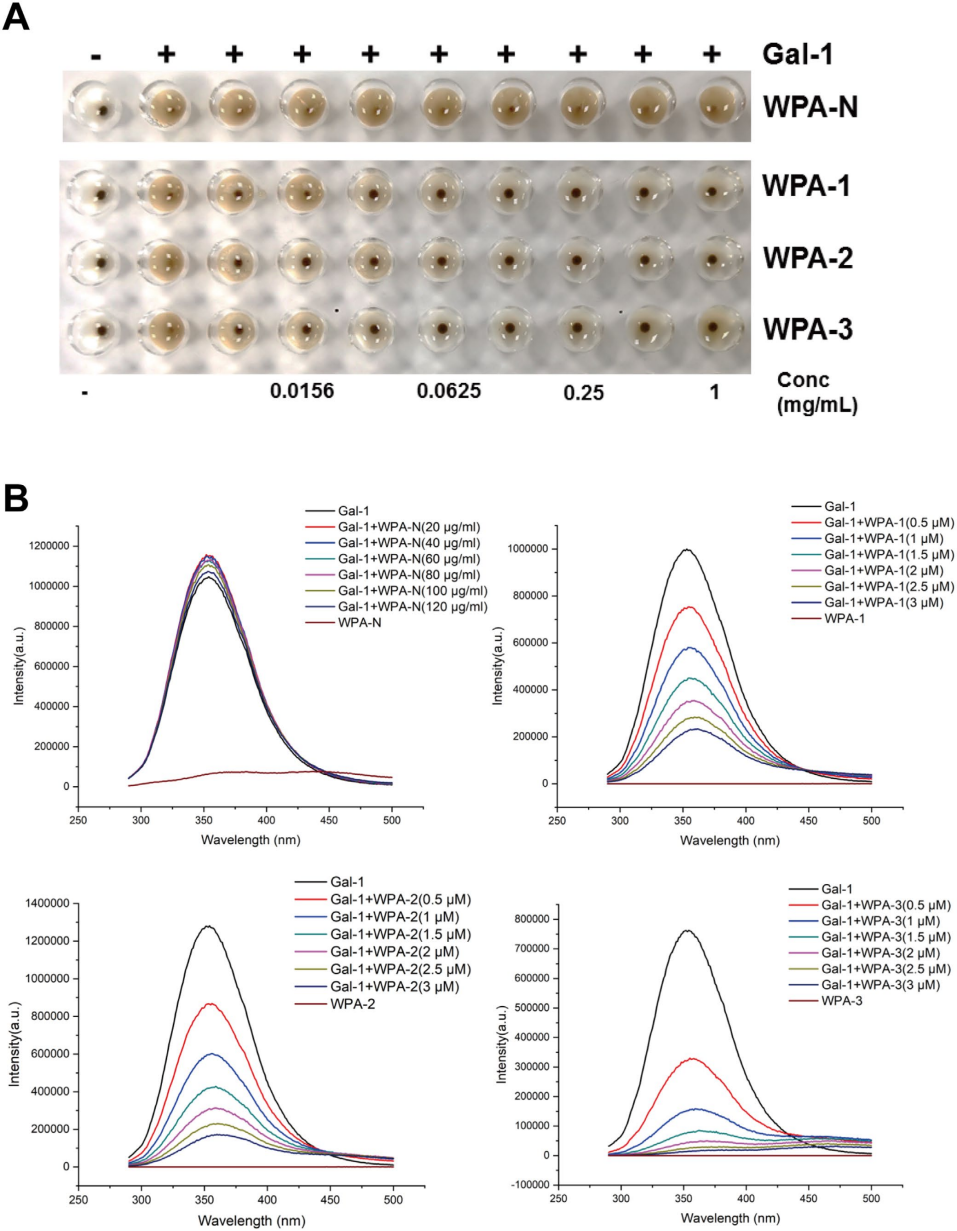
The inhibitory activity of WPA fractions on Gal‐1. (A) Gal‐1‐mediated hemagglutination inhibited by WPA fractions; (B) Fluorescence intensity of Gal‐1 inhibited by WPA fractions.

There are many different oligosaccharide chains on the surface of red blood cell membranes, which can recognize corresponding proteins and bind to them, causing red blood cells to agglutinate. If an inhibitor is added to the system, the inhibitor competitively binds to the corresponding protein, and the protein can no longer bind to the oligosaccharide chains on the surface of the red blood cell membrane, and the red blood cells no longer agglutinate. The lower the concentration of the required saccharide for complete inhibition of red blood cell agglutination, the stronger the binding ability between the saccharide and Gal‐1. The minimum inhibitory concentration (MIC) can measure the strength of the interaction between the saccharide and Gal‐1. The results of WPA fractions inhibiting Gal‐1‐mediated hemagglutination were shown in Figure 4A. WPA‐1, WPA‐2, and WPA‐3 can all bind to Gal‐1, with WPA‐3 having the strongest binding ability (MIC = 7.8 μg/mL), while WPA‐N was unable to inhibit Gal‐1‐mediated hemagglutination at a maximum concentration of 4 mg/mL.

The Gal‐1‐mediated hemagglutination inhibitory activity of WPA fractions was further confirmed by the fluorescence spectroscopy method, and the results were shown in Figure 4B. When WPA fractions inhibit Gal‐1, the fluorescence spectrum can provide information on whether the Trp residues in Gal‐1 are bound and inhibited. Gal‐1 can generate a corresponding fluorescence spectrum when excited at 280 nm due to the presence of Trp residues. If there is an interaction between polysaccharides and Gal‐1, it can change the internal Trp residue microenvironment of Gal‐1, resulting in weakened fluorescence intensity of Gal‐1. Gal‐1 can generate fluorescence intensity after being excited at the 280 nm wavelength. With the gradual increase of different concentrations of polysaccharides, it was found that the fluorescence intensity of Gal‐1 gradually weakened with the increase of WPA‐1, WPA‐2, and WPA‐3 concentrations, and the fluorescence intensity attenuation degree of WPA‐3 was the largest. With the increase of concentration, the fluorescence intensity of Gal‐1 almost did not change for WPA‐N. The above results indicated that there was an interaction between WPA‐1, WPA‐2, WPA‐3, and Gal‐1, and the interaction between WPA‐3 and Gal‐1 was the strongest, while the interaction between WPA‐N and Gal‐1 was the weakest. Wu et al. (2020) and Gao et al. (2013) reported that galactan was not the only important factor in the interaction between pectin and galectin, but also Ara residues played important roles. Thus, among the WPA fractions, WPA‐3 showed the strongest activity, probably owing to the sum content of Gal and Ara in WPA‐3 being much higher than in other fractions.

### 3.5 Antitumor Activity of WPA Fractions

**FIGURE 5:**
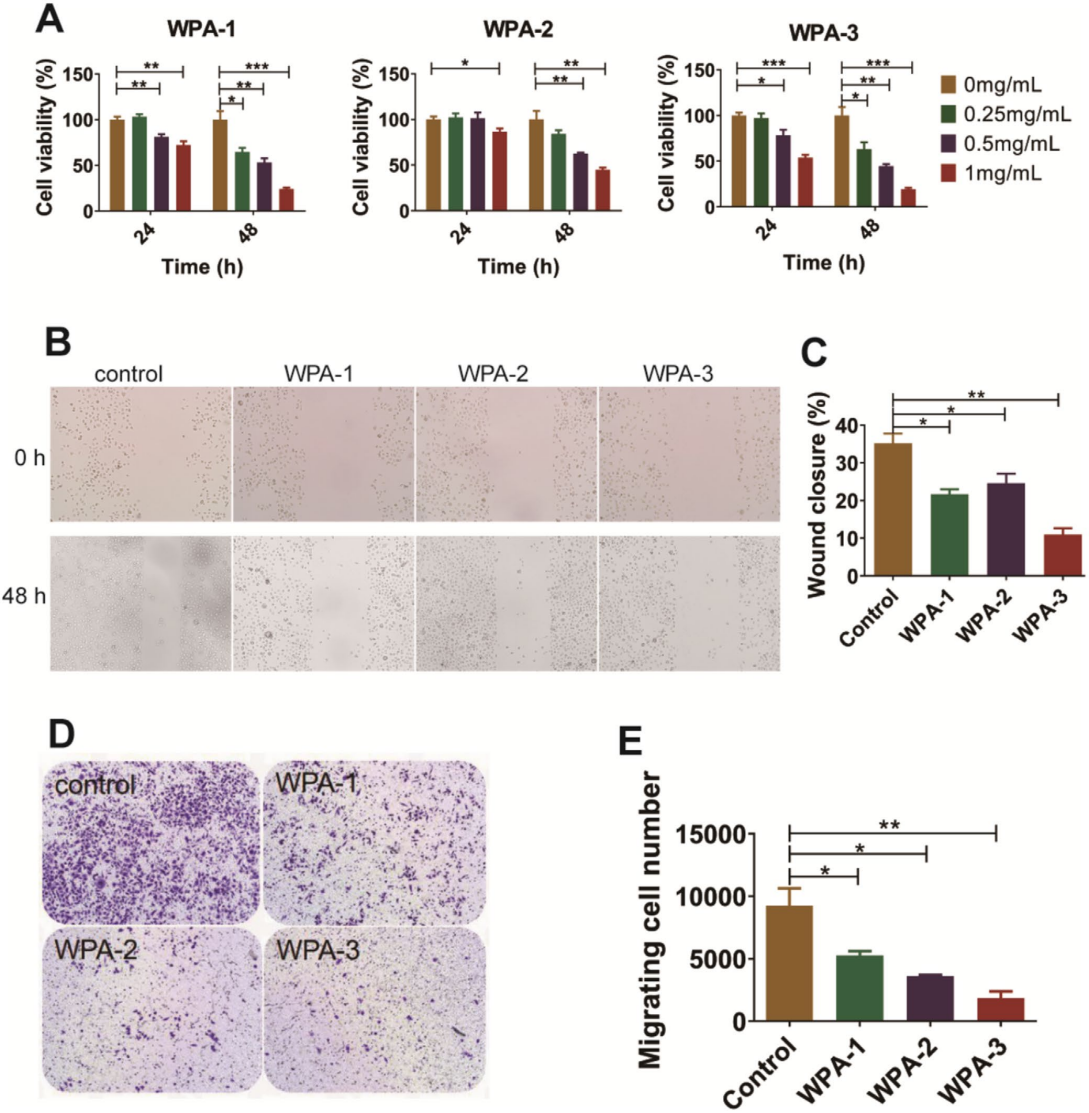
Antitumor activity of WPA fractions in vitro. Inhibitory activity of WPA fractions on ME180 cell proliferation (A), migration by scratch assay (B), and migration by transwell assay (C) ($\bar{x}$ ± SD, *n* = 3). *p < 0.05；**p < 0.01; ***p < 0.001.

Previous studies have confirmed that high expression of Gal‐1 is associated with invasion and metastasis in cervical cancer significantly, and it is also an independent premarker of poor specific survival in cervical cancer patients who will receive postoperative radiotherapy treatment more frequently (Astorgues‐Xerri et al. 2014; Chetry et al. 2020; Huang et al. 2013; Kohrenhagen et al. 2006). Therefore, cervical cancer cell line ME180 was used in this study to analyze the antitumor activity of WPA fractions based on antagonizing Gal‐1 function. The antiproliferative activities of three acidic fractions (WPA‐1, WPA‐2, and WPA‐3) were analyzed on cervical cancer cell lines ME180 in vitro. As shown in Figure 5A, the viabilities of ME180 cells were significantly inhibited by WPA‐1, WPA‐2, and WPA‐3 in a concentration‐dependent manner at 48 h, and these three fractions showed cell viability in this order: WPA‐3 (19.5%) > WPA‐1 (24.2%) > WPA‐2 (45.0%). The inhibitory activities of three acidic fractions (WPA‐1, WPA‐2, and WPA‐3) on the migration of ME180 cells were analyzed using both scratching assay and transwell assay. As shown in Figure 5B,C, for scratching assay, photos and quantitative analyses both showed that the migration of ME180 cells was inhibited by WPA‐1, WPA‐2, and WPA‐3 treatment at 48 h. Three acidic fractions showed wound closure in the order: WPA‐3 (11.0%) > WPA‐1 (21.6%) > WPA‐2 (24.6%). As shown in Figure 5D,F, the results of ME180 cell migration in the transwell assay were consistent with the results in the scratching assay, and WPA‐3 showed the strongest activity with a calculated migrating cell number value of 1858. Among the acidic fractions, WPA‐3 showed the strongest antitumor activity on ME180 cells, which is probably related to its higher content of Gal residues, a key monosaccharide that binds to Gal‐1. Fan et al. (2010) also found that ginseng pectin fractions WGPA‐3‐RG and WGPA‐4‐RG showed stronger inhibitory activities than WGPA‐3‐HG and WGPA‐4‐HG on L‐929 cell migration, which was related to WGPA‐3‐RG and WGPA‐4‐RG both containing more Gal residues.

**FIGURE 6:**
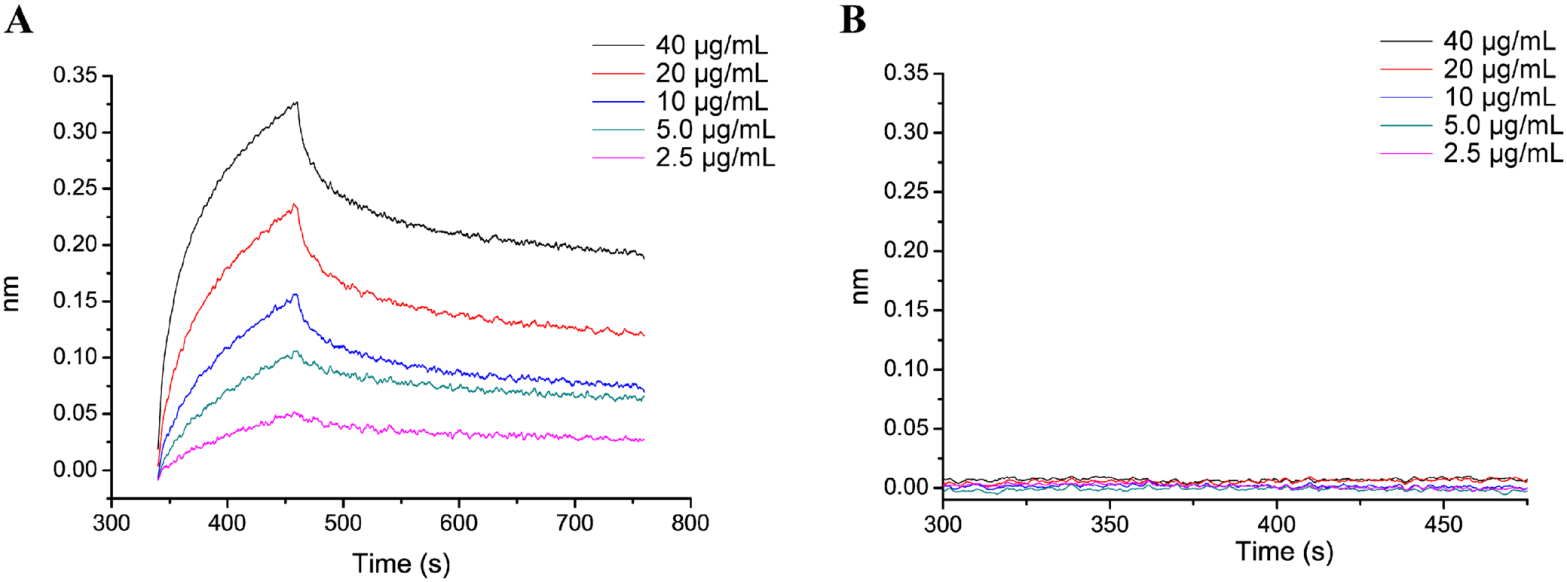
BLI sensorgram displayed the change of binding response (in nm) between five concentrations of WPA‐3 (2.5, 5, 10, 20, 40 μg/mL) and immobilized Gal‐1. Association and dissociation signals were shown (A). Nonspecific binding of WPA‐3 to Ni‐NTA sensor (B).

The activity results obtained above indicated that WPA‐3 was the most active fraction; therefore, BLI technology was used to analyze the binding affinity between WPA‐3 and Gal‐1. BLI, a label‐free technique, is commonly used to analyze the interaction between biomolecules, and the dissociation constant (*KD*) is calculated by association rate and dissociation rate values using several sample concentrations. As shown in Figure 6, the association signals and dissociation signals were displayed, which suggested that the affinity of WPA binding to Gal‐1 enhanced with increasing WPA‐3 concentrations in a concentration‐dependent manner. After being calculated by BLI software, the *KD* value of WPA‐3 was 26.8 nM, suggesting WPA‐3 binding affinity to Gal‐1 was very strong.

**FIGURE 7:**
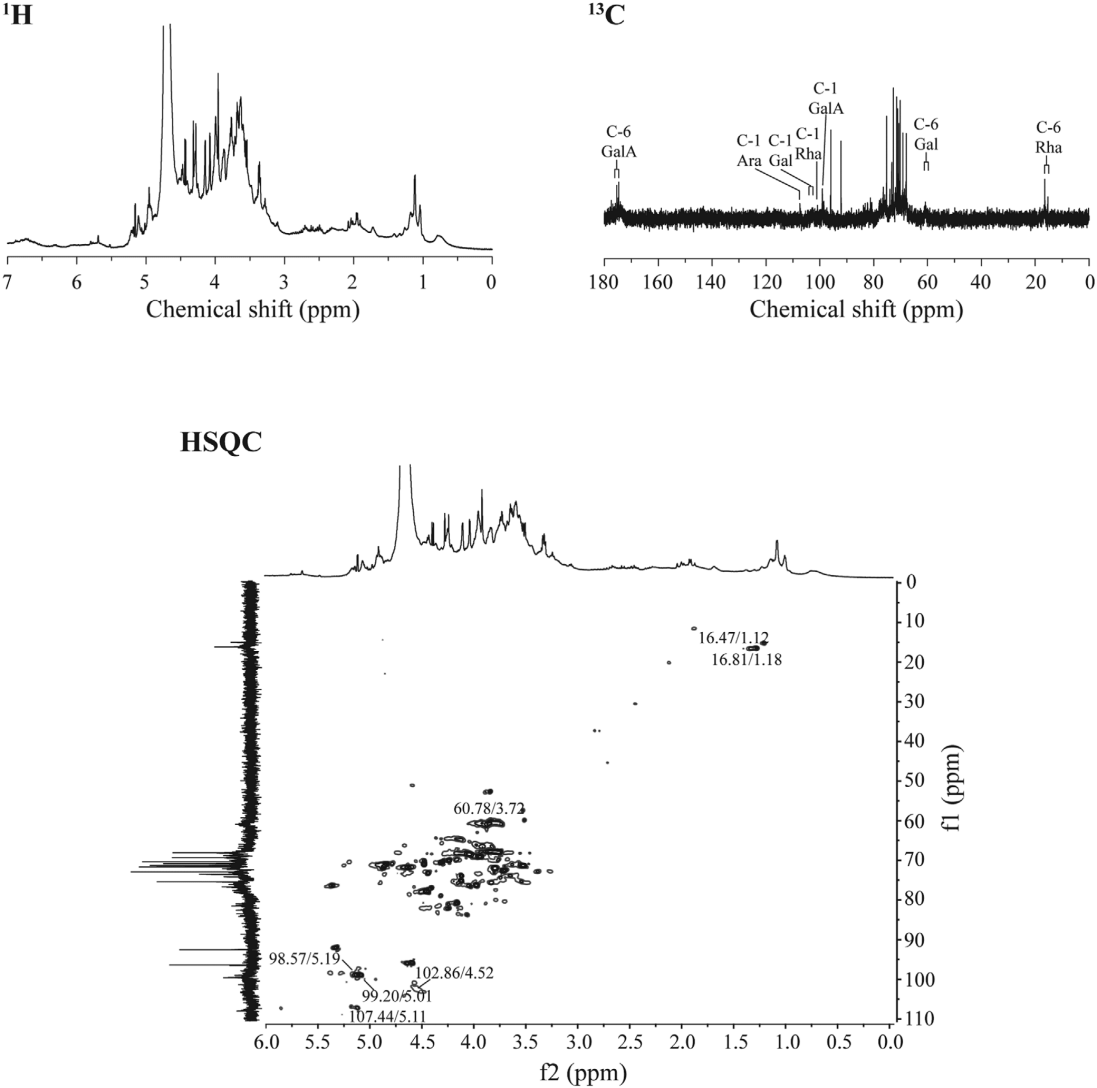
One‐dimensional NMR spectra (^1^H, ^13^C) and two‐dimensional NMR spectra (HSQC) of WPA‐3.

The activity of pectic polysaccharides is closely related to their primary structure, such as monosaccharide molar ratio, molecular mass, glycosidic bond, etc. (Yu et al. 2018). Thus, the further structural feature of WPA‐3 was analyzed by NMR, and the spectra were shown in Figure 7. The NMR spectra of WPA‐3 had Rha residues, with resonances at 98.57/5.19 ppm, 76.44/4.08 ppm, 69.14/3.81 ppm, 71.90/3.32 ppm, 68.58/3.68 ppm, and 16.47/1.12 ppm, corresponding to the C1/H1 to C6/H6 groups from [→2)‐α‐Rha‐(1→]_*n*_ units, and it also had GalA groups, with resonances at 99.20/5.01 ppm, 67.90/3.63 ppm, 68.10/3.99 ppm, 77.84/4.32 ppm, 70.16/− ppm, and 175.46/– ppm, corresponding to the C1/H1 to C6/H6 groups from [α‐(1 → 4)‐GalA]_*n*_ units (Figure 7) (Yu et al. 2010; Zhang, Zu, et al. 2020; Zhang, Liu, et al. 2020; Zhang, Shuai, et al. 2020; Zhang et al. 2009). Besides, the resonances of covalent bonding between Rha and GalA from [→2)‐α‐Rha‐(1 → 4)‐GalA→]_*n*_ units were also observed. In addition, WPA‐3 contained notably some monosaccharide residues, such as Ara, Gal, and Glc, so the C/H resonances from Ara, Gal, and Glc groups were also obtained. For Ara groups, the resonances at 107.44/5.11 ppm, 82.31/4.28 ppm, 85.63/4.03 ppm, 83.85/4.28 ppm, and 69.14/3.88 ppm were assigned to C1/H1 to C6/H6 groups from [α‐(1 → 3.5)‐Ara]_*n*_ units, respectively. For Gal groups, the resonances at 104.29/4.52 ppm and 102.86/4.52 ppm were assigned to C‐1/H‐1 from β‐(1 → 3.6)‐Gal units and β‐(1 → 3)‐Gal units, respectively. Based on the data above, WPA‐3 might contain a rhamnogalacturonan‐I backbone with branched galactan, arabinan, and arabinogalactans‐II side chains and some homogalacturonan domains.

## 4 Conclusions

In conclusion, four purified water‐soluble polysaccharide subfractions, including a neutral polysaccharide fraction (WPA‐N) and three acidic polysaccharide fractions (WPA‐1, WPA‐2, and WPA‐3) were purified from *P. asiatica* . WPA‐N was mainly composed of Glc and Gal, while WPA‐1, WPA‐2, and WPA‐3 were mainly constituted by GalA, Gal, Rha, Ara, etc. Except for WPA‐N, the three acidic fractions could all inhibit Gal‐1‐mediated hemagglutination and the fluorescence intensity of Gal‐1, and they could also inhibit the proliferation and migration activity of ME180 cells with high expression Gal‐1 significantly. Among the fractions, WPA‐3 showed the strongest activity, with a *KD* value of 26.8 nM analyzed by the biolayer interferometry method. The primary structure of WPA‐3 could be defined as rhamnogalacturonan‐I main chain with branched galactan, arabinan, and arabinogalactans‐II side chains and some homogalacturonan domain. Therefore, we hypothesized that Gal‐1 might be an important potential target for the anti‐cervical cancer activity of WPA‐3. In the future, we will further investigate the molecular mechanisms of the anti‐cervical cancer activity of WPA‐3. Meanwhile, these results provide valuable information on the structure of *P. asiatica* polysaccharide and its potential application in functional food fields.

## Author Contributions

**Yiqing Li:** data curation (lead), methodology (lead), writing – original draft (lead). **Yonghong Ma:** data curation (equal), methodology (equal). **Guanyu Li:** investigation (equal), supervision (equal). **Yanqing Jia:** investigation (equal), supervision (equal). **Chengxin Sun:** methodology (equal), writing – review and editing (equal). **Liushaoqiu Zhou:** software (equal), validation (equal), visualization (equal). **Wenqin Lei:** software (equal), validation (equal), visualization (equal). **Tao Zhang:** conceptualization (lead), methodology (lead), writing – review and editing (lead).

## Conflicts of Interest

The authors declare no conflicts of interest.

## Data Availability

Data will be made available on request.
